# Genome Sequence of Mycobacterium abscessus Phage phiT46-1

**DOI:** 10.1128/MRA.01421-20

**Published:** 2021-01-14

**Authors:** Elizabeth D. Amarh, Rebekah M. Dedrick, Rebecca A. Garlena, Daniel A. Russell, Deborah Jacobs-Sera, Graham F. Hatfull

**Affiliations:** a Department of Biological Sciences, University of Pittsburgh, Pittsburgh, Pennsylvania, USA; Loyola University Chicago

## Abstract

Mycobacteriophage phiT46-1 is a newly isolated *Mycobacterium* phage that was isolated by spontaneous release from Mycobacterium abscessus strain Taiwan-46 and infects M. abscessus strain BWH-C. Phage phiT46-1 is unrelated to previously described mycobacteriophages, has a 52,849-bp genome, and includes a polymorphic toxin-immunity cassette associated with type VII secretion systems.

## ANNOUNCEMENT

Mycobacterium abscessus is a nontuberculous mycobacterium (NTM) that is ubiquitous in the environment and is common in water and soil (1). M. abscessus is a common cause of pulmonary and disseminated infections in immunocompromised individuals, particularly those with cystic fibrosis (2, 3). Antibiotic treatment of NTM infections is challenging, with widespread multidrug resistance and nonresponsiveness to antibiotic therapy (4). Mycobacteriophages are viruses that infect mycobacterial hosts and, although many have been isolated on Mycobacterium smegmatis, few infect M. abscessus (5). Isolation and characterization of M. abscessus phages will advance our understanding of M. abscessus and their potential therapeutic utility (5).

Many strains of M. abscessus carry prophages (6–9) and are expected to spontaneously release phage particles. Phage phiT46-1 was isolated by plating a culture supernatant of M. abscessus Taiwan-46 (provided by Chidiebere Akusobi and Eric Rubin) onto a lawn of M. abscessus BWH-C on solid medium at 37°C, using standard methods (10). Phage were picked from infected areas, plaque purified, and amplified on BWH-C, and DNA was extracted by phenol-chloroform-isoamyl alcohol extraction (10). A sequencing library was prepared from genomic DNA using a NEBNext Ultra II FS kit with dual-indexed barcoding and was included as one of a pool of 48 phage genome libraries on an Illumina MiSeq system, yielding 931,342 paired-end 300-base reads and 2,400-fold coverage of the phiT46-1 genome. These reads were assembled using Newbler v2.9 with default settings, yielding a single 52,849-bp contig with a G+C content of 64%. The contig was evaluated for completeness and accuracy using Consed v29. Sequencing read alignments did not identify unique genome ends, and either there are multiple distinct termini or the contig is circularly permuted (11); for genome representation, it was linearized with coordinate 1 at the first codon of the small terminase subunit gene. Phage phiT46-1 is not closely related to other actinobacteriophages (nucleotide identities span <4% of the total genome length [12]) but shares several virion structural genes with cluster Q phages (13). phiT46-1 does not infect M. smegmatis.

The programs GeneMarkS v4.30 (14), Glimmer v3.02 (15), Phamerator Actino_prophage v5 (16), and DNA Master v5.23.5 (http://cobamide2.bio.pitt.edu) were used to identify 78 protein-coding genes in the phiT46-1 genome. All tools were run with default parameters unless otherwise specified. The genome has no tRNA genes, as indicated by ARAGORN v1.2.41 (17). Of the 78 predicted genes, 45% were assigned putative functions using BLAST (18) and HHpred (19, 20). The virion structure and assembly genes suggest that phiT46-1 has a siphoviral morphology (family *Siphoviridae*), and repressor and tyrosine integrase genes are consistent with its temperate nature. The predicted early lytic genes also include an HNH endonuclease, a phosphoadenosine phosphosulfate (PAPS) reductase, an oxidoreductase, WhiB, and a RecET-like recombination system.

**FIG 1:**
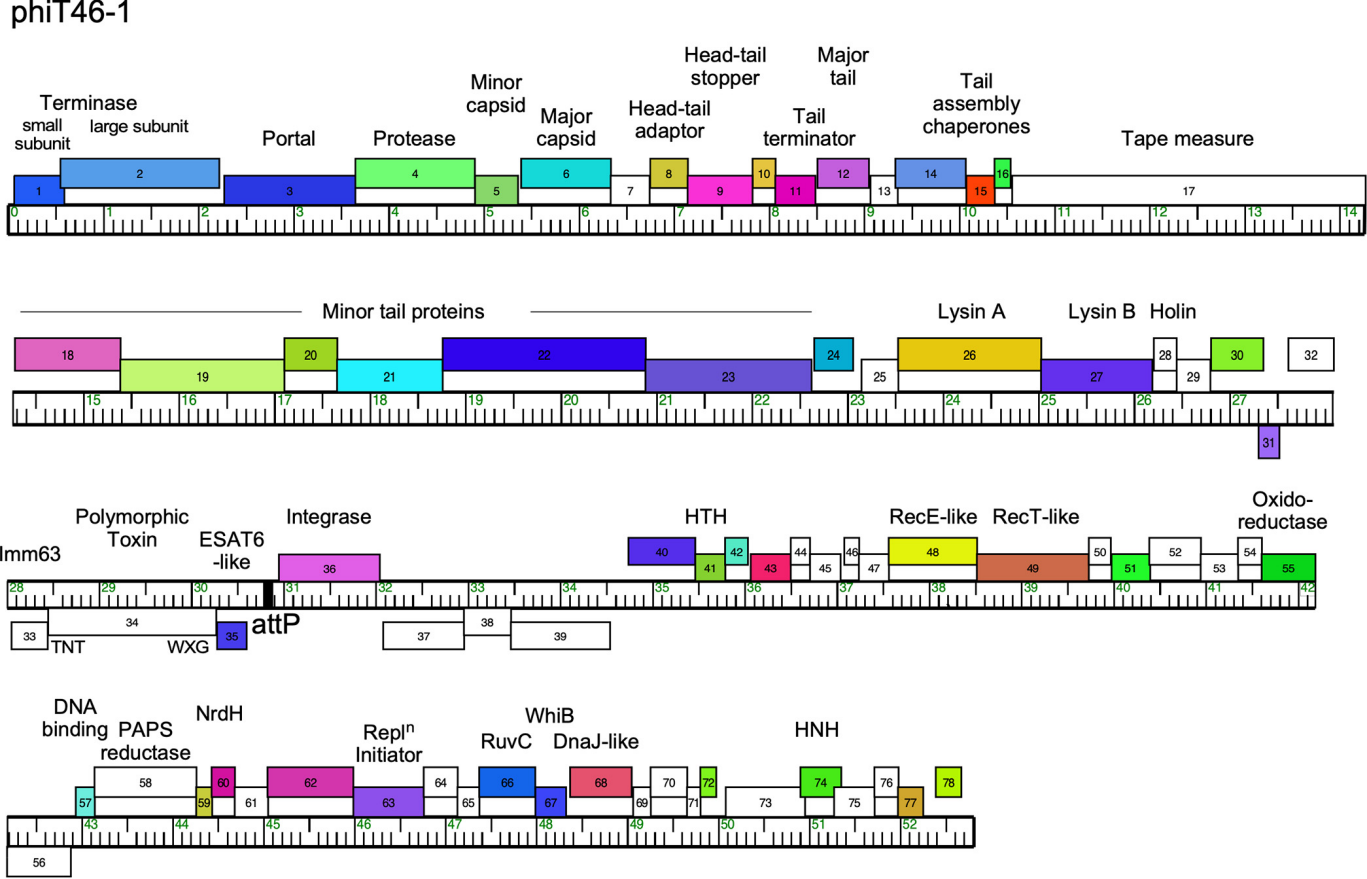
Genome organization of phage phiT46-1. The linearized viral genome of phage phiT46-1 is shown with genes represented by colored boxes either above or below the genome, reflecting rightward and leftward transcription, respectively; gene numbers are shown in each box. Genes are colored according to the “phamily” designations (16), with white boxes representing “orphams” with no close relatives in this data set. Phamily assignments were determined using Phamerator (16) and the Actino_prophage database (version 5). Predicted gene functions are indicated.

Interestingly, phiT46-1 contains a cassette coding for a polymorphic toxin (PT), an immunity (Imm63) protein, and an ESAT-6-like protein (Fig. 1). The 50-kDa PT has an N-terminal WXG-100 motif (21) and a C-terminal domain containing a tuberculosis necrotizing toxin (TNT) (22). The genomic location of the cassette, adjacent to the integration apparatus, suggests that these genes may be lysogenically expressed (23), likely with secretion via a type VII secretion pathway (24). The TNT motif is associated with escape of M. tuberculosis from phagosomes, and phiT46-1 is thus implicated in the survival of M. abscessus T46 in infected human cells.

### Data availability.

The phiT46-1 sequence is available in GenBank with accession no. MW353181, and sequencing reads are available with accession no. SRX9186031.
